# N and N′-mediated recognition confers resistance to tomato brown rugose fruit virus

**DOI:** 10.17912/micropub.biology.000660

**Published:** 2022-10-29

**Authors:** Antoine Pelletier, Peter Moffett

**Affiliations:** 1 Centre SÈVE, Département de Biologie, Université de Sherbrooke, Sherbrooke, Québec, Canada

## Abstract

Tomato brown rugose fruit virus (ToBRFV) is an emerging tobamovirus that overcomes the *Tm-2 ^2^* resistance gene used in commercial tomato plants to protect against tobamoviruses. In this article, we show that ToBRFV is recognised through its P50 replicase fragment by the resistance gene *N* in *N. tabacum* , which triggers a hypersensitive response (HR). We also demonstrate that the *N’* gene provides protection against ToBRFV through recognition of the viral coat protein without triggering a typical HR in *N. tabacum* .

**Figure 1. (A) N. tabacum cultivars Samsun NN (NN), Samsun nn (nn), BY-2 and W38 were rub-inoculated with TMV or ToBRFV, as indicated, on two leaves. Photographs were taken 4 and 19 days post-inoculation (DPI), as indicated, to show local and systemic symptoms, respectively. (B) Presence or absence of viral transcripts in local and systemic tissues from (A) was assessed by RT-PCR using virus-specific primers (virus), as indicated. RNA quality was assessed by RT-PCR using primers specific to the L25 gene transcript. (C) The four cultivars used in (A) were infiltrated with Agrobacterium tumefaciens carrying binary vectors expressing the P50 fragment, the coat protein (CP), and the movement protein of ToBRFV (MP), as well as the TMV P50 fragment, the ToMV CP and an empty vector (EV), as indicated in the schematic diagram. All six infiltration spots in N. benthamiana leaves were co-infiltrated with an extra strain carrying a binary vector expressing the N’ protein. N. tabacum and N. benthamiana leaves were photographed at 5 and 10 DPI, respectively.**
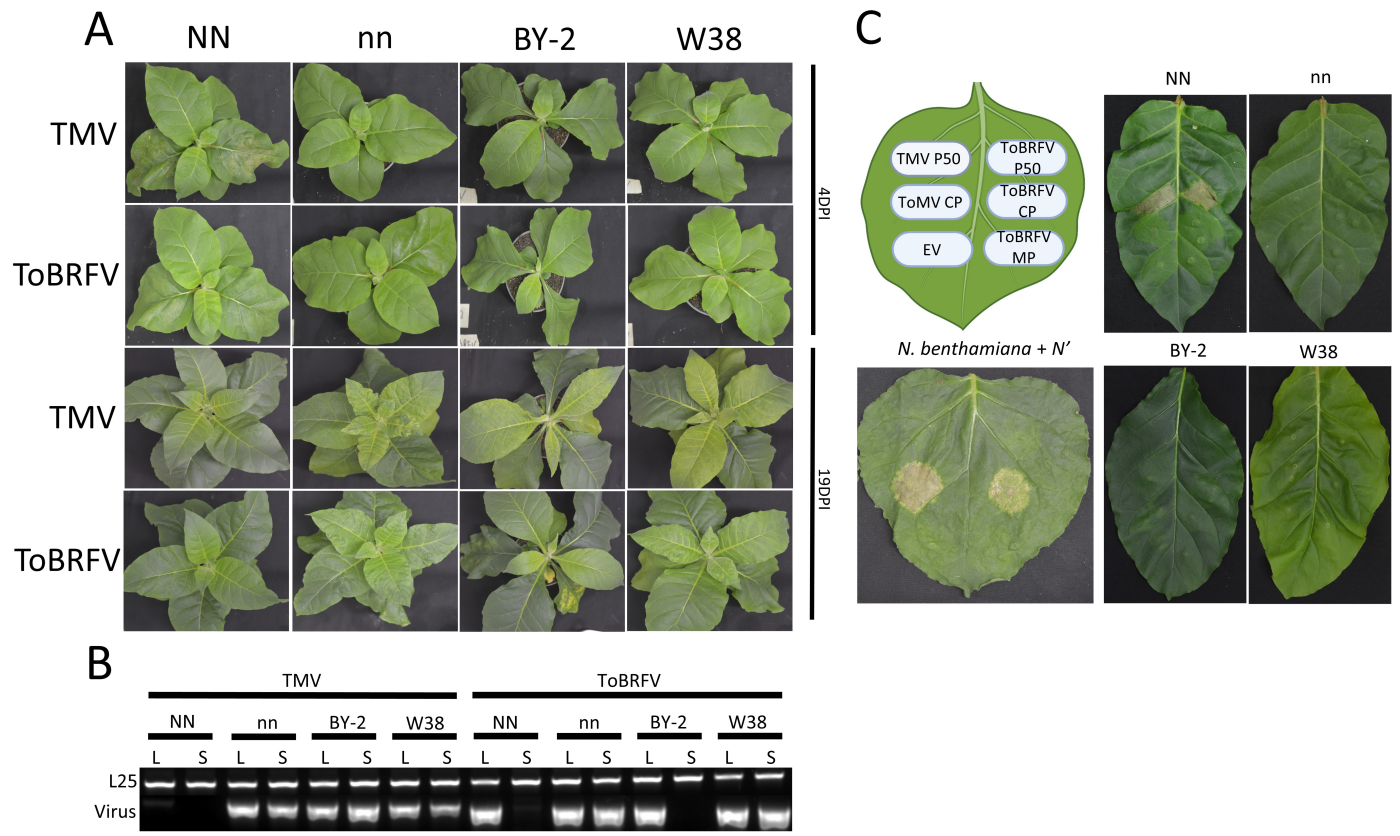
Resistance to ToBRFV in tobacco

## Description

Members of the virus genus *Tobamovirus* are composed of single stranded positive RNA encapsulated in a cylindrical shaped particle composed of coat protein (CP) subunits assembled around the genomic RNA (Ishibashi and Ishikawa 2016). To defend against pathogens, plants encode large numbers of disease resistance (R) genes that code for nucleotide-binding leucine-rich repeat (NLR) proteins, which recognize specific pathogen-encoded proteins. Activation of these receptors by the latter induces a defense response that can result in localised cell death, known as the hypersensitive response (HR) (Dalio et al. 2021). Tomato brown rugose fruit virus (ToBRFV) was reported in 2014 (Salem et al. 2016; Luria et al. 2017) and has rapidly become an important threat to greenhouse tomato cultivation, transmitted via contaminated seed and mechanical contact (Zhang et al. 2022). The tomato *Tm-2 ^2^* gene confers resistance to most tomato-infecting tobamoviruses through recognition of tobamoviral movement proteins (MP) (Weber and Pfitzner 1998). However, changes in the ToBRFV MP allow it to overcome this resistance, albeit at the cost of slower cell-to-cell movement, resulting in attenuated systemic infection (Yan et al. 2021; Hak and Spiegelman 2021). In addition to *Tm-2 ^2^* , several other solanaceous R genes confer resistance to tobamoviruses, including the tobacco *N* and *N’* genes, which recognize the tobamoviral P50 replicase fragment and CP, respectively (Sekine et al. 2012; Erickson et al. 1999).

To test if ToBRFV is recognized by tobacco tobamoviral-recognizing NLRs, we used four different *N. tabacum* cultivars, including W38, which contains no tobamovirus-specific R gene. Samsun *NN* expresses the *N* resistance gene that confers resistance to tobacco mosaic virus (TMV), whereas Samsun *nn* does not. BY-2 possesses the *N’* resistance gene whose gene product recognises the CP of multiple tobamoviruses, including that of ToMV, but very few TMV strains (Sekine et al., 2012). Plants were rub-inoculated with sap containing TMV or ToBRFV and photographed at 4 days post-inoculation (DPI) and 19 DPI to document local and systemic infection progression, respectively. Four days after inoculation with TMV or ToBRFV, little to no symptoms were apparent on the cultivars W38, Samsun *nn* and BY-2, apart for some light chlorosis in ToBRFV-inoculated BY-2 and Samsun *nn* (Fig 1A). At 19 DPI, both viruses had caused systemic infections in the cultivars Samsun *nn* and W38, as evidenced by mottling and chlorosis in newly emerged, non-inoculated leaves. As expected, inoculation of the BY-2 cultivar with TMV did not result in HR lesions on inoculated leaves and did result in systemic chlorosis and mottling (Fig 1A). In contrast, ToBRFV inoculation on BY-2 leaves resulted in chlorotic lesions rather than the necrotic HR lesions typically seen in Samsun *NN* (Fig. 1A). Nonetheless, no systemic symptoms were observed in systemic BY-2 leaves 19 days after inoculation with ToBRFV, indicating that this cultivar is resistant to ToBRFV (Fig. 1A). Leaves of Samsun *NN* inoculated with TMV or ToBRFV showed HR lesions on locally infected leaves and no signs of infection in systemic leaves were observed, indicating that the *N* resistance gene prevented the spread of the virus throughout the plants. The presence or absence of viral transcripts in local and systemic tissues was validated by RT-PCR amplification of the CP coding sequence (Fig. 1B). Consistent with the observed symptoms, robust amplification of viral RNA was detected in systemic tissues of W38 and Samsun *nn* , but not in BY-2 or Samsun *NN* (Fig. 1B). A very weak amplification of ToBRFV was observed in Samsun *NN* systemic tissues, possibly due to residual virions originating from infected leaves or simply due to the ease of contamination of ToBRFV through mechanical handling (Zhang et al. 2022). Nonetheless, given the combination of infection and RT-PCR results, we conclude that the *N* and *N’* genes effectively confer resistance to ToBRFV.

To complement infection assays, ToBRFV proteins were expressed in leaves of the different tobacco cultivars using *Agrobacterium tumefaciens* -mediated transient expression (agroinfiltration). Expression of the P50 fragments from both TMV and ToBRFV induced strong HR reactions in Samsun *NN* plants at 5DPI (Fig. 1C), indicating that the P50 replicase fragment of ToBRFV is recognized by the N protein in the same way as the P50 fragment from TMV. In contrast, transient expression of ToMV or ToBRFV CP did not induce HR in BY-2 leaves. Although this is unexpected, given the resistance to ToBRFV observed in the BY-2 (Fig 1A), co-expression of NLRs and the proteins they recognize, including Tm-22 and the TMV MP, does not always result in an HR in all species (Bhattacharjee et al. 2009). Recognition of tobamoviral CP was thus validated by transient co-expression in *N. benthamiana* . In this assay, co-expression of *N’* with the TMV or ToBRFV CP, but not the MP or P50 proteins, resulted in an HR (Fig 1C), thus demonstrating that the N’ protein does indeed recognize the CP of these viruses.

We conclude that both the *N* and *N’* genes can confer resistance to ToBRFV through recognition of the viral P50 fragment and CP, respectively. Although resistance is achieved in both cases, it appears that *N’* mediated resistance does not induce a typical HR response in *N. tabacum* . These results demonstrate that genetically encoded resistance to ToBRFV exists based on typical NLR-encoding genes, indicating the potential for discovering or engineering resistance to this virus in tomato.

## Methods

Plant and virus material

*Nicotiana benthamiana* and *Nicotiana tabacum* were grown in soil (BM6, Berger,) in growth chambers with a 12h dark, 12h light photoperiod at 22°C.

ToBRFV-infected tomato leaf samples were ground in 0.1M phosphate buffer (2mL g ^-1^ of infected tissue) to produce a sap that was used to infect *N. benthamiana.* Additional sap was produced in the same manner with infected *N. benthamiana* tissue and was used for all experiments.

Virus inoculation

6-week-old *N. tabacum* plants were inoculated using the sap produced from infected tissues and applied to two leaves on each plant along with silicon carbide (Alfa Aesar, 320 grit).

Plasmid construction

RNA extraction was performed on ToBRFV-infected tissues followed by reverse transcriptase (RT) using mmuLV reverse transcriptase to obtain cDNA, from which individual ToBRFV ORFs were amplified using specific primers with a 5’ XbaI and a 3’ SalI overhang. Inserts were than cloned into the pBIN61 plasmid with a 3’ HA tag (Sacco et al. 2007) linearized with XbaI and SalI using T4 DNA ligase. P50 expression plasmid was previously described in Bhattacharjee et al. 2009 and ToMV CP expression vector was described in Hamel et al. 2016.

Transient expression

Binary vectors were transformed into *Agrobacterium tumefaciens* C58C1 carrying the virulence helper plasmid pCH32 (Bendahmane et al. 1999) and used for agroinfiltration as previously described (Hamel et al. 2016). Individual strains were diluted to an optical density of 0.15 before being infiltrated into *N. tabacum* or *N. benthamiana* leaves using a needleless syringe.

## Reagents

**Table:** 

Table 1. Plant cultivar and bacterial strains used in this study		
**Strain**	**Genotype**	**Source**
*N. tabacum*	*N. tabacum* Samsun *NN*	USDA GRIN
*N. tabacum*	*N. tabacum* Samsun *nn*	USDA GRIN
*N. tabacum*	*N. tabacum* BY-2	USDA GRIN
*N. tabacum*	*N. tabacum* W38	USDA GRIN
*N. benthamiana*	*N. benthamiana* WT	Baulcombe Laboratory
C58C1 + virulence helper plasmid pCH32	*Agrobacterium tumefaciens*	Baulcombe Laboratory (Bendahmane et al. 1999)

**Table:** 

Table 2. Plasmids used in this study		
**Plasmid**	**Description**	**Source**
pBIN61 (TMV P50)	Binary expression vector for TMV large replicase component under CaMV 35s promoter	Bhattacharjee et al. 2009
pBIN61 (ToMV CP)	Binary expression vector for ToMV CP under CaMV 35s promoter	Hamel et al. 2016
pBIN61 (EV)	Binary expression vector with empty cloning site under CaMV 35s promoter	Hamel et al. 2016
pBIN61 (ToBRFV P50)	Binary expression vector for ToBRFV large replicase component under CaMV 35s promoter	This study
pBIN61 (ToBRFV CP)	Binary expression vector for ToBRFV CP under CaMV 35s promoter	This study
pBIN61 (ToBRFV MP)	Binary expression vector for ToBRFV movement protein under CaMV 35s promoter	This study
pBIN61 (N’-HA)	Binary expression vector for *N’* gene under CaMV 35s promoter.	Hamel et al. 2016

**Table:** 

Table 3. Primers used in this study			
**Target**	**Forward sequence**	**Reverse sequence**	**Purpose**
ToBRFV large replicase fragment	cctaggtctagagccaccatgGAAATAGAGTCATTAGAGCAATTCC	ATTGCTGTCGACatattgggtccctgcatc	Cloning viral sequences into pBIN61 Identification of viral transcripts in infected tissue RT-PCR
ToBRFV CP	cctaggtctagaatgtcttacacaatcgcaactc	gctatagtcgacagaagatgcaggtgcag	Cloning viral sequences into pBIN61
ToBRFV movement protein	cctaggtctagaatggctcttgttaagggtaaag	gctatagtcgacaaAATACGAATCTGAATCGGCG	Cloning viral sequences into pBIN61
*N. tabacum* L25	cctccgtttcttcagcaacttc	ttttggccaacatccaactcac	Identification of *N. tabacum* reference gene in infected tissue RT-PCR
TMV-U1	TCGAATTCAATATGTCTTACAGTATC	CCGTTCTAGATTATGCATCTTGACT	Identification of viral transcripts in infected tissue RT-PCR
